# Best clinical practice guidance for treating deep carious lesions in primary teeth: an EAPD policy document

**DOI:** 10.1007/s40368-022-00718-6

**Published:** 2022-10-11

**Authors:** M. Duggal, S. Gizani, S. Albadri, N. Krämer, E. Stratigaki, H. J. Tong, K. Seremidi, D. Kloukos, A. BaniHani, R. M. Santamaría, S. Hu, M. Maden, S. Amend, C. Boutsiouki, K. Bekes, N. Lygidakis, R. Frankenberger, J. Monteiro, V. Anttonnen, R. Leith, M. Sobczak, S. Rajasekharan, S. Parekh

**Affiliations:** 1 College of Dental Medicine, QU Health, Qatar University, Doha, Qatar; 2 Department of Paediatric Dentistry, School of Dentistry, National and Kapodistrian, University of Athens, Athens, Greece; 3 School of Dentistry, Unit of Oral Health, University of Liverpool, Liverpool, UK; 4 Department of Paediatric Dentistry, Justus-Liebig University Gießen, Giessen, Germany; 5 Department of Pediatric Oral Health and Orthodontics, University Center of Dental Medicine, Basel, Switzerland; 6 Discipline of Orthodontics and Paediatric Dentistry, Faculty of Dentistry, National University of Singapore, Singapore, Singapore; 7 Department of Orthodontics and Dentofacial Orthopedics, School of Dental Medicine, University of Bern, Bern, Switzerland; 8 Department of Orthodontics and Dentofacial Orthopedics, 251 Hellenic Air Force and VA General Hospital, Athens, Greece; 9 Department of Paediatric Dentistry, School of Dentistry, University of Leeds, Leeds, UK; 10 Department of Preventive and Paediatric Dentistry, University of Greifswald, Greifswald, Germany; 11 Faculty of Dentistry, National University of Singapore, Singapore, Singapore; 12 Liverpool Reviews and Implementation Group, University of Liverpool, Liverpool, UK; 13 Department of Paediatric Dentistry, Justus-Liebig-University Giessen, University Medical Centre Giessen and Marburg (Campus Giessen) Medical Centre for Dentistry, Schlangenzahl 14, 35392 Giessen, Germany; 14 Department of Paediatric Dentistry, Medical University Vienna, University Clinic of Dentistry, Sensengasse 2a, 1090 Vienna, Austria; 15 Lygidakis Dental Clinic (Private Dental Practice), 2 Papadiamantopoulou str. & Vasilissis Sofias Ave, 11528 Athens, Greece; 16 Medical Centre for Dentistry, Department of Operative Dentistry and Endodontics, Phillips-University Marburg, University Medical Centre Giessen and Marburg (Campus Marburg), Georg-Voigt-Str. 3, 35039 Marburg, Germany; 17 Department of Paediatric Dentistry, Sheffield Teaching Hospitals, Sheffield, UK; 18 Research Unit of Oral Health Sciences, University of Oulu, Oulu, Finland; 19 Dublin Dental University Hospital, Trinity College, Dublin, Ireland; 20 Specialized Dental Practice, Warsaw, Poland; 21 Department of Paediatric Dentistry, School of Oral Health Sciences, Ghent University, B-9000 Ghent, Belgium; 22 Department of Paediatric Dentistry, UCL Eastman Dental Institute, London, UK

**Keywords:** Primary molars, Caries, Caries management, Minimal intervention dentistry, Pulp treatment, Dental materials

## Abstract

**Purpose:**

The European Academy of Paediatric Dentistry (EAPD) has developed this best clinical practice guidance to help clinicians manage deep carious lesions in primary teeth.

**Methods:**

Three expert groups conducted systematic reviews of the relevant literature. The topics were: (1) conventional techniques (2) Minimal Intervention Dentistry (MID) and (3) materials. Workshops were held during the corresponding EAPD interim seminar in Oslo in April 2021. Several clinical based recommendations and statements were agreed upon, and gaps in our knowledge were identified.

**Results:**

There is strong evidence that indirect pulp capping and pulpotomy techniques, and 38% Silver Diamine Fluoride are shown to be effective for the management of caries in the primary dentition. Due to the strict criteria, it is not possible to give clear recommendations on which materials are most appropriate for restoring primary teeth with deep carious lesions. Atraumatic Restorative Technique (ART) is not suitable for multi-surface caries, and Pre-formed Metal Crowns (PMCs) using the Hall technique reduce patient discomfort. GIC and RMGIC seem to be more favourable given the lower annual failure rate compared to HVGIC and MRGIC. Glass carbomer cannot be recommended due to inferior marginal adaptation and fractures. Compomers, hybrid composite resins and bulk-fill composite resins demonstrated similar values for annual failure rates.

**Conclusion:**

The management of deep carious lesions in primary teeth can be challenging and must consider the patient’s compliance, operator skills, materials and costs. There is a clear need to increase the use of MID techniques in managing carious primary teeth as a mainstream rather than a compromise option.

## Aim

The European Academy of Paediatric Dentistry (EAPD) proposes this best-practice guidance to help practitioners manage deep caries in children during the delivery of oral health care. A similar statement has been published by a Joint ORCA and EFCD Expert Delphi Consensus Statement (Spleith et al. 2020). Treatment options and materials for permanent teeth are excluded from this document.

## Selection of the topic guide

Dental caries is a common, yet preventable disease that affects 20–90% of 6 year-old children in Europe (WHO 2018). The management of dental caries in children has shifted towards controlling caries according to an individual treatment plan including risk estimation, early diagnosis and prevention plan to keep dentition healthy and arrest initial lesions if needed (Pitts et al 2014). This was investigated by the EAPD best clinical guidance management for early caries lesions in children and young adults (Kühnisch et al. 2016).

Unfortunately, many children may present with deep carious lesions which require restorative management, either by conventional techniques or by implementing the concept of Minimal Intervention Dentistry (Ericson et al. 2003; Frecken et al. 2012; Dorri et al. 2015; Schwendicke et al. 2019). Conventional approaches to deep carious lesions have focussed on pulpal interventions to avoid extraction and keep the tooth asymptomatic and functional until exfoliation, whereas Minimal Intervention Dentistry (MID) techniques aim to maintain teeth vital, asymptomatic and functional for as long as possible, preferably until exfoliation. The type of treatment provided should follow biological evidence-based caries management concepts, which emphasise preserving as much tooth structure as feasible, and in case of primary teeth, until these exfoliate naturally (Frencken et al. 2012). For all these techniques, the clinician must also consider the most suitable material to use.

When a clinician is presented with a child patient with deep carious lesions in the primary teeth, there are many factors to be considered before an appropriate management plan can be reached. These need to consider both the needs of the patient, parent and dentist:

### Patient factors:

- General health of the child
- Dental status
- Patient co-operation
- Signs and symptoms of pulpitis
- Cavity size (extent and activity of the lesion)

### Parent factors:

- Parent expectations (motivation and compliance)
- Cost

### Dentist factors:

- Clinician competence
- Materials available

With a move towards MID vs. conventional restorative methods, it can be confusing to know which technique to use. This guidance aims to provide clinicians with the best evidence-based recommendations for treating deep carious lesions in primary teeth where available or to recommend good clinical practice where evidence is weak.

## Methods

Three expert groups were invited by EAPD to undertake systematic reviews of the available literature for the management of deep carious lesions in primary teeth, in particular focussed on:

- Conventional management (systematic review, Stratigaki et al. 2022)
- MID (umbrella review, BaniHani et al. 2021)
- Materials (systematic review, Amend et al. 2022)

This new guideline is based on the reviews presented by the invited experts in the 12th EAPD virtual interim meeting in Oslo in April 2021. The discussions were carried out by those attending the three working groups consisting of invited speakers and nominated delegates from the EAPD member countries. Each working group was moderated by two members of the EAPD Clinical Affairs Committee (CAC).

- Conventional management: M Duggal, S Gizani, E Stratigaki, HJ Tong, K Seremidi, D Kloukos, (Moderators: J Monteiro, E Stratigaki)
- MID: S Albadri, R Santamaria, A BaniHani, S Hu, M Maden (Moderators: V Anttonen, R Leith)
- Materials: N Krämer, K Bekes, S Amend, C Boutsiouki, D Kloukos, N Lygidakis, R Frankenberger (Moderators: M Sobczak, S Rajasekharan)

Discussions were carried out and conclusions were reached by agreement and consensus, and the recommendations from the workshops were presented on the final day of the interim meeting by the CAC moderators. This was used as a basis by the CAC members to develop the guidance.

The selection criteria for the three groups is summarised in (Table 1). Due to the different selection criteria and approaches used in the three reviews, it was not possible to determine recommendations using GRADE (Guyatt et al. 2008). This implies that some of the recommendations are based on low-grade evidence and expert opinion.

**Table 1:** Selection criteria of the three reviews undertaken

Name	Review type	Inclusion criteria	Exclusion criteria	Follow up	Outcomes
Conventional	Systematic review with meta-analysis	Children and adolescents with deep caries in vital primary molars Local or general anaesthesia Rubber dam isolation	Permanent teeth Irreversible pulpitis	At least 24 months	Clinical success Radiographic success
MID	Umbrella review of systematic reviews (with & without meta-analysis)	Children with untreated carious lesion(s) extending into dentine (ICDAS 4 & 5) in primary teeth No dentine carious tissue removal Non restorative cavity control (NRCC) Selective or stepwise caries removal	Caries removal was assisted by chemomechanical agents Use of local anaesthesia and rubber dam	At least 6 months	Symptom free vital tooth maintained until exfoliation Caries arrest
Materials	Systematic review	Primary teeth treated by vital pulp therapy or endodontic treatment RCTs Lesions extending into dentine requiring intervention	Permanent teeth Drop-out rate > 30%	At least 12 months Minimum of 40 restorations per group	Modified USPHS criteria Assessment of restoration quality

## Results

### Workshop 1: conventional management of deep caries in primary molars

The systematic review and meta-analysis by Stratigaki et al. 2022, concentrated on the following techniques:

- Direct Pulp Capping
- Indirect Pulp Capping
- Pulpotomy
- Pulpectomy

The evidence demonstrated that pulp reaction to the treatment and applied medicament rely on the status of the pulp before the intervention, and the conditions under which the pulp is being treated (patient’s compliance, effective use of Local Anaesthesia (LA), and Rubber Dam Isolation).

### Recommendations:

- Use the least invasive technique for the best predictable clinical outcome.
- There was a unanimous agreement that a restoration providing a good coronal seal is essential for the management of vital pulp in primary teeth.
- Indirect Pulp Capping (IPC) and Pulpotomy (PP) have high success rates and can be recommended as effective treatment modalities for the management of deep caries in primary teeth.
- Direct pulp capping has limited use in daily clinical practice in the event of pulp exposure, except in very restricted non-infectious conditions and on asymptomatic teeth.
- Calcium hydroxide has the poorest success rate of all commonly used pulpotomy medicaments, and therefore it is recommended that calcium hydroxide should not be considered as a material suitable to be used as a pulpotomy medicament.
- Formocresol (FC), Ferric Sulphate (FS) and Mineral Trioxide Aggregate (MTA) all demonstrate similar success rates when used as pulpotomy medicaments. Given that concerns have been expressed regarding the potential toxic effects of certain medicaments, such as formocresol, it is recommended that clinicians should use alternatives, such as FS or MTA that have similar reported outcomes.
- Pulpectomy is not recommended as a first line of treatment for deep caries management of vital primary molars, due to the existence of more conservative successful alternatives. Nevertheless, pulpectomy may be considered over extraction in certain situations where tooth loss would compromise the child’s dental health and long-term occlusion (i.e., minimise space loss) or such as in the absence of a permanent successor.
- Clinicians should consider clinical success as a primary indicator of a successful outcome, rather than considering further interventions based on radiographic failure alone.

## Gaps in knowledge:

- More studies are needed to compare medicaments within the same technique
- Further comparison studies are needed between techniques with longer follow-up rates
- More studies are needed to compare irrigation disinfectant medicaments for pulp and surrounding tissues

### Workshop 2: minimal intervention dentistry

Evidence provided by the umbrella review by BaniHani et al., 2021 was used to consider the usability of the following MID techniques for managing deep carious lesions in primary teeth:

- The use of 38% Silver Diamine Fluoride (SDF)
- The use of pre-formed metal crowns (PMCs) using the Hall Technique
- Selective (one step) and step-wise caries removal
- The use of Atraumatic Restorative Technique (ART)

### Recommendations:

- The use of 38% SDF once or twice per year can be advantageous for caries arrest, with better outcomes for two applications per year. It is recommended that clinicians should consider the use of 38% SDF in children with a high caries risk, to avoid/delay the need for more invasive treatments in very young children.
- The use of pre-formed metal crowns (PMC) using the Hall technique (HT) for the management of dentinal caries in primary teeth can reduce the risk of pain and restoration failure for caries in the primary teeth. The Hall technique (HT) reduced discomfort and was preferred by patients and parents.
- Selective (one-step) or step-wise caries removal offer some advantage over complete caries removal for the avoidance of pulp exposure for lesions extending to inner third or quarter of dentine. In the absence of other signs and symptoms indicating irreversible pulpitis, these techniques should be considered to avoid pulp exposure and the need for pulp therapy.
- The failure rates for ART when used to restore multi-surface caries is unacceptably high. Therefore, this technique is not recommended for the restoration of multi-surface carious lesions. ART could be considered as an adequate management option for single surface (occlusal) in certain instances for primary teeth.

## Gaps in knowledge:

- Further investigation is needed into the effectiveness and safety of the HT, as there has only been one systematic review to date.
- Comparison studies are needed into the cost effectiveness of different MID treatments modalities

### Workshop 3: materials

The systematic reviews by Amend et al. 2022 was considered by the working group for the following materials:

- Amalgam
- Glass Ionomer cements (GIC)
- Glass carbomers
- Compomers
- Composites
- Full coverage crowns

Within the parameters chosen for the review, it was determined there was no evidence from well-designed, randomised clinical studies in children available to determine which materials are most effective for deep caries in primary teeth. This implies that most of the recommendations are based on low-grade evidence and expert opinion.

### Recommendations:

- The European Academy of Paediatric Dentistry based on Minamata Convention do not recommend the further use of Amalgam in the restoration of primary teeth (Minamata 2013).
- Due to the low evidence studies (high or unclear risk of bias), additional considerations regarding application technique (such as use of disinfectant, cavity conditioner before material placement or bilayer technique or coating) could not be considered.
- Glass Ionomer Cements (GIC), High Viscosity GIC (HVGIC) and Resin Modified Glass Ionomer Cements (RMGIC) are recommended for occlusal (class I) restorations in primary teeth.
- High Viscosity GIC (HVGIC) and Resin Modified Glass Ionomer Cements (RMGIC) are recommended with caution for occluso-proximal (class II) restorations of primary dentition. These materials are not recommended in multi-surface reconstructions.
- Metal Reinforced GIC (MRGIC) are not recommended in the restoration of primary molars.
- Glass Carbomer is not recommended for both occlusal (class I) and occluso-proximal (class II) restorations of primary carious molars due to the high failure rate.
- Compomers are recommended for both occlusal (class I) and occluso-proximal (class II) restorations of primary carious molars.
- Hybrid and bulk-fill composite resins are recommended for both occlusal (class I) and occluso-proximal (class II) restorations of primary carious molars.
- It is recommended to use a calibrated polymerization lamp and ensure adequate polymerization, omitting the monomers at the surface.
- Due to lack of evidence, it was not possible to consider dentine etching times and margin cavity preparation.
- Due to the selection criteria, only one RCT with Preformed Metal Crowns (PMC) was included in the review, using the Hall Technique (HT) in vital primary molars (Santamaria et al. 2017). The study was found to have a low annual failure rate, but a high risk of bias, therefore clear recommendations could not be given due to lack of evidence.
- There is a lack of RCTs evaluating restoration techniques in primary anterior teeth. In the one included trial with high risk of bias (Alaki et al. 2020) zirconia crowns and composite strip crowns were compared in the reconstruction of carious primary anterior teeth, but a recommendation could not be given for lack of evidence.
- Due to the low levels of evidence, no recommendations for the use of specific isolation techniques could be made for all dental materials.

## Gaps in knowledge:

- More RCTs with power calculations and parallel group design are needed comparing restorative interventions
- Narrow age range for included children and longer follow ups
- A description of the caries experience among the included participants
- Detailed descriptions of the interventions (availability of preoperative radiographs, assessment of carious lesion depth, administration of local anaesthesia, isolation technique, extent of carious tissue removal, restorative materials and application mode, adhesive protocol etc.)
- Operator experience should be clearly stated
- A precise report of the numbers of patients lost to follow-up is essential

### Clinical recommendations

**Fig. 1:**
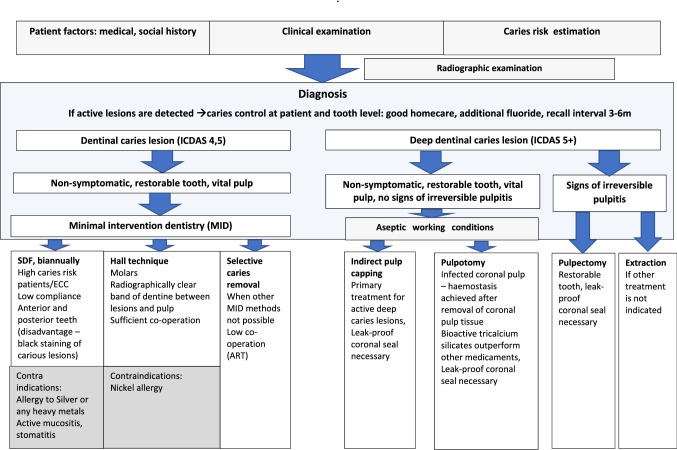
Flowchart of treatment protocol of dentinal caries lesions in primary dentition

Recommendations for management of deep carious lesions in primary teeth were developed in line with the strength of the evidence (Fig. 1).

#### Strong

- It is recommended that application of 38% SDF can be advantageous for caries arrest, with better outcomes for biannual application.
- Indirect Pulp Capping (IPC) or selective and step-wise caries removal, and Pulpotomy (PP) have high success rates and can be recommended as effective treatment modalities for the management of deep caries in primary teeth.
- The use of formocresol for pulpotomy is no longer recommended, due to the availability of more biocompatible medicaments.
- ART technique is not recommended for the restoration of multi-surface carious lesions.
- Glass carbomer cannot be recommended due to inferior marginal adaptation and fractures.
- MRGIC cannot be recommended due to loss of anatomical form and marginal intergrity.
- Pre-formed metal crowns (PMCs) using the Hall technique are recommended as a treatment option for the management of dentinal caries.
- The use of PMCs for endodontically treated primary molar teeth is recommended.

#### Weak

- Compomers are recommended for both occlusal (class I) and occluso-proximal (class II) restorations of carious primary molars.
- Hybrid and bulk-fill composite resins are recommended for both occlusal (class I) and occluso-proximal (class II) restorations of carious primary molars.
- GIC, RMGIC and HVGIC are more favourable given the lower annual failure rate compared to MRGIC.

### Research recommendations

The EAPD interim seminar identified further research needs, to improve comparability of studies to include:

- Focus conducting trials with more appropriate study designs and standardised methodology, particularly in relation to use of randomisation and allocation sequence concealment diagnostic and outcome measures
  o Studies should record the use of radiographic assessment
  p The depth of caries should be specified using an objective classification such as that proposed by the ICDAS
- Quality of life, patient preference, cost effectiveness and burden of care and impact of different treatments modalities on future compliance

## Conclusion

The management of deep carious lesions in primary teeth can be challenging and must consider the patient’s compliance, operator skills, materials and costs. The lack of high quality RCTs meant that for some consensus statements only a low level of evidence was available.

One of the important outcomes of this review was that Minimal Intervention Dentistry (MID) techniques appear to be effective in arresting the progression of dentinal caries in primary teeth when compared to no treatment and conventional restorations. There is some evidence of improved patients reported outcomes with such techniques, however further research is required. A major advantage of MID for the management of dentine carious lesions is that many of these techniques can be carried out without aerosol generation. There is a clear need to increase the emphasis on utilising MID techniques in managing carious primary teeth as a mainstream rather than compromise option in circumstances where the conventional approach is prohibited due to cost or co-operation (Splieth et al, 2020).

Due to the heterogenicity of the studies and the reviews, it was not possible to develop guidance using best-practice methods, such as GRADE. Detailed and explicit criteria for ratings of quality and grading of strength, as well as consensus protocols, and input from patients and parents will make judgments more transparent for future guideline development and recommendations.
